# Targeted Metabolomic Profiling Reveals Association Between Altered Amino Acids and Poor Functional Recovery After Stroke

**DOI:** 10.3389/fneur.2019.01425

**Published:** 2020-01-24

**Authors:** Xin Wang, Tao Liu, Haixin Song, Shaoyang Cui, Gang Liu, Andrea Christoforou, Patrick Flaherty, Xun Luo, Lisa Wood, Qing Mei Wang

**Affiliations:** ^1^Stroke Biological Recovery Laboratory, Spaulding Rehabilitation Hospital, Boston, MA, United States; ^2^Department of Rehabilitation, Clinical Medical College, Yangzhou University, Yangzhou, China; ^3^Clinical School of Acupuncture, Moxibustion and Rehabilitation, Guangzhou University of Chinese Medicine, Guangzhou, China; ^4^Department of Mathematics, College of Science and Mathematics, University of Massachusetts Boston, Boston, MA, United States; ^5^Kerry Rehabilitation Medicine Research Institute, Shenzhen, China; ^6^William F. Connell School of Nursing, Boston College, Chestnut Hill, MA, United States

**Keywords:** ischemic stroke, metabolomics, recovery, arginine, mass spectrometry, amino acids

## Abstract

Amino acids have been shown to be among the most important metabolites to be altered following stroke; however, they are a double-edged sword with regard to regulating hemostasis. In this study, we conducted a targeted metabolomic study to examine the association between serum levels of amino acids and functional recovery after stroke. Three hundred and fifty-one patients with stroke admitted to an acute rehabilitation hospital were screened, and 106 patients were selected based on inclusion and exclusion criteria. Recruited patients were stratified using Montebello Rehabilitation Factor Score (MRFS) efficiency. We selected the top (*n* = 20, 19%) and bottom (*n* = 20, 19%) of MRFS efficiency for metabolomic analysis. A total of 21 serum amino acids levels were measured using ultra high performance liquid chromatography and mass spectrometry. The normalized data were analyzed by multivariate approaches, and the selected potential biomarkers were combined in different combinations for prediction of stroke functional recovery. The results demonstrated that there were significant differences in leucine-isoleucine, proline, threonine, glutamic acid, and arginine levels between good and poor recovery groups. In the training (0.952) and test (0.835) sets, metabolite biomarker panels composed of proline, glutamic acid, and arginine had the highest sensitivity and specificity in distinguishing good recovery from poor. In particular, arginine was present in the top 10 combinations of the average area under the receiver operating characteristic curve (AUC) test set. Our findings suggest that amino acids related to energy metabolism and excitotoxicity may play an important role in functional recovery after stroke. Therefore, the level of serum arginine has predictive value for the recovery rate after stroke.

## Introduction

Increasing evidence suggests that amino acids, including homocysteine and branched-chain amino acids (BCAA), are one of the most important disturbed metabolites after stroke (1, 2). Importantly, recent studies suggest that amino acids could have beneficial and detrimental effects. For example, glutamate plays an important role in maintaining the normal signal transduction of nerve cells, which is beneficial to the synaptic plasticity of neurons and to the recovery of stroke (3). However, elevated levels of glutamate can trigger oxidative stress, inflammation, and endothelial damage (4). BCAAs were also found to be low in stroke patients compared with normal controls, and lower BCAA levels correlated with poor neurological outcome in stroke patients (5). On the other hand, higher concentrations of baseline BCAA were associated with increased risk of stroke in a high cardiovascular risk population (6). Therefore, elucidating the effects of dysregulated amino acid levels on stroke recovery will contribute to identifying prognostic biomarkers and formulating effective therapeutic interventions. However, there are few studies on the relationship between the changes in amino acid levels and the rate of functional recovery after stroke (7, 8).

Metabolomics is a new strategy that can detect changes in amino acids, vitamins, organic acids, and other small-molecule metabolites in biofluids (e.g., plasma or serum) of patients in real time (9, 10). As it is difficult to detect the metabolites directly in the brain, metabolites in serum are usually used as alternative indicators to reflect biological and pathological functions of the brain (11, 12). Metabolic alterations in the brain can result in changes in the metabolome of biofluids (12, 13), especially those metabolites with low molecular weight, which may be easily exchanged through the meningo between the cerebrospinal fluid (CSF) and the blood (5, 14). As a result, potential biomarkers associated with stroke recovery can be detected in serum by metabolomics, and signaling pathways involved in stroke recovery can be drawn.

Currently, most studies of metabolomic biomarkers in patients with stroke compare metabolomic profiles of stroke patients with the normal population (9, 13). Few studies explore the differences in metabolomic biomarkers among stroke patients with good or poor functional recovery (4). Therefore, in this study, we performed a metabolomic analysis of serum amino acid levels in stroke patients undergoing acute inpatient rehabilitation. Our initial hypothesis was that alterations in amino acids can affect the rate of functional recovery in stroke patients. The aim of this study was to find new biomarkers with high sensitivity and specificity, and to provide a basis for further investigation into the rehabilitation mechanism of stroke, as well as the development of targeted treatment methods and improvement of prognosis.

## Materials and Methods

### Study Participants

**Figure 1:**
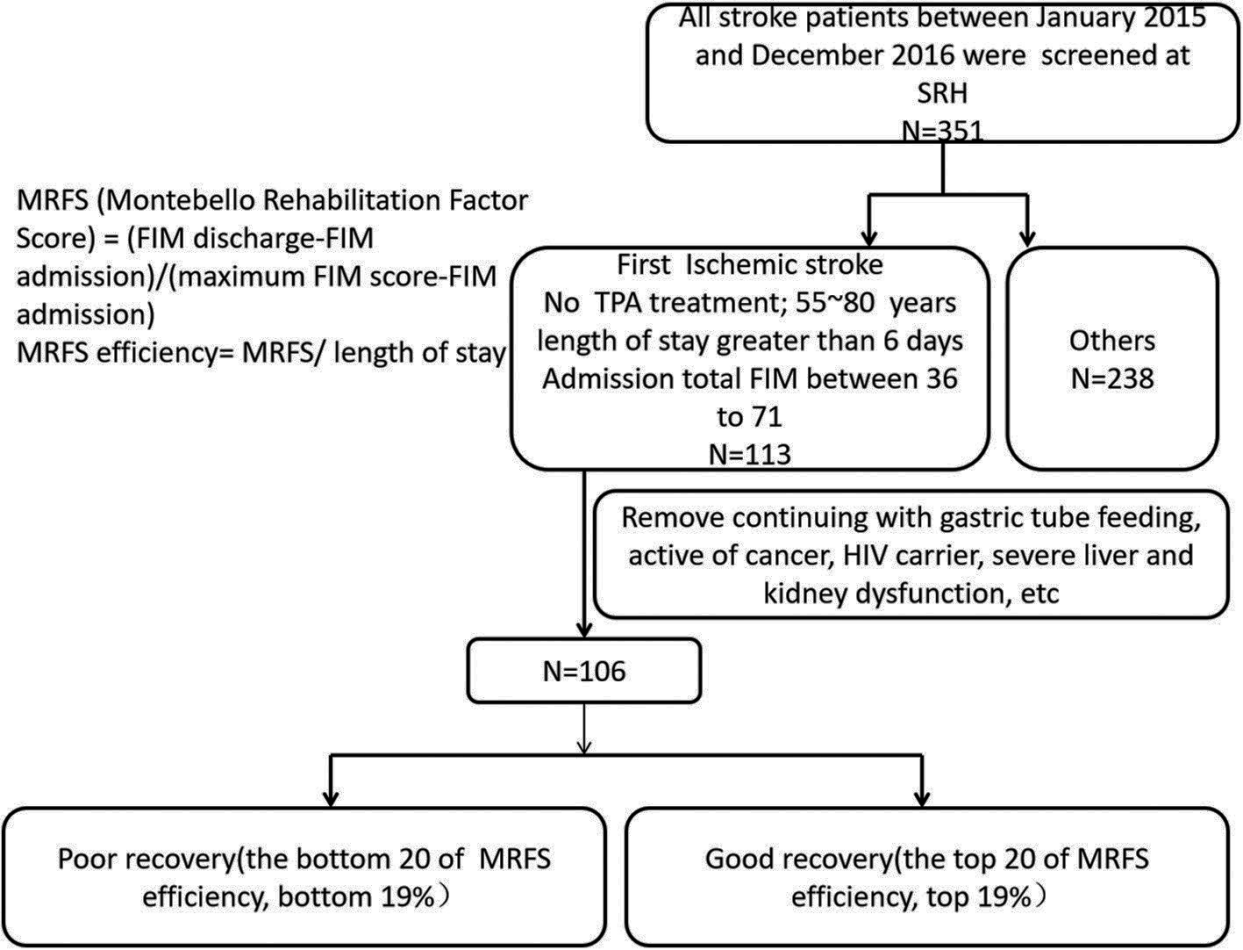
Patients with stroke were screened according to the inclusion criteria and the exclusion criteria.

This is a retrospective study approved by the ethics committee of Spaulding Rehabilitation Hospital. Three hundred and fifty-one patients with stroke admitted to an acute rehabilitation hospital in Boston between January 2015 and December 2016 were screened with the following inclusion criteria: first ischemic stroke, confirmed by CT or MRI, no tPA (tissue plasminogen activator) treatment, age between 50 and 85 years, length of stay >6 days, admission total Functional Independence Measure (FIM) score between 36 and 71 (12, 15, 16), and has serum samples available upon admission. Exclusion criteria included: continuing with gastric tube feeding, active cancer, HIV (human acquired immunodeficiency) carrier, or severe liver and kidney dysfunction (12, 15, 16). All patients received standard inpatient rehabilitation including physical therapy, occupational therapy, and speech therapy. One hundred and six patients met the inclusion/exclusion criteria and were stratified at the top 20 of MRFS efficiency (top 19%) and the bottom 20 of MRFS efficiency (bottom 19%), and they were defined as good recovery group (GR) and poor recovery group (PR) (Figure 1). The MRFS efficiency formula is described in the following section.

### Functional Independence Measure Scale

FIM scale is widely used to evaluate the functional abilities of stroke patients undergoing rehabilitation (15, 17). The scale includes 18 items graded on a 7-point ordinal scale, with a maximum total score of 126 where a lower score means less functional independence. The total FIM scores were recorded at admission and discharge. The gain of the total FIM score, which is commonly used to evaluate functional recovery after stroke, was determined for every patient by calculating the difference of the total FIM score from admission to discharge (15, 17).

Because length of stay and total FIM scores at admission varied considerably, Montebello Rehabilitation Factor Score (MRFS) efficiency was used to evaluate functional recovery after stroke (17, 18). The MRFS evaluates relative gain, and this method depends on the validated FIM score. According to this method, the basis for calculating relative gain is a patient's specific potential for improvement (maximal possible FIM – actual admission FIM). The actual score ranges from 0 to 1, and the MRFS can overcome the misinterpretation of the ceiling effect. The MRFS was calculated using the following formula: MRFS = (discharge FIM – admission FIM)/(maximum FIM score – admission FIM). MRFS efficiency reflects recovery and functional outcomes of stroke more appropriately and precisely because it is measured relative to the potential for change and overcomes the fact that different patients have different admission FIM scores (15, 18). MRFS efficiency = MRFS/length of stay (15, 17, 18).

### Clinical Characteristics

Demographic data and clinical characteristics including age, gender, education, body mass index (BMI), diagnosis, comobilities, and discharge destination were collected from electronic medical records. Stroke features including site (supratentorial and infratentorial) and side were collected from MRI or CT reports. Lesion size was not measured due to limited imaging available for the measurement.

### Sample Preparation

Non-fasting venous blood was obtained from all stroke patients within 1 week after hospitalization. Studies showed that there were no significant difference of amino acids between fasting serum samples and non-fasting serum samples (19, 20). The blood sample testing process follows standard protocol of ultra high performance liquid chromatography—mass spectrometry (UHPLC-MS), and the standard protocol are as follow.

Blood samples were centrifuged at 13,000 g for 30 min at 4°C, after which time serum was removed and aliquotted before storage at −80°C (not more than 2 years) until ultra high performance liquid chromatography—mass spectrometry (UHPLC-MS) analysis. Prior to analysis, serum samples were thawed and centrifuged at 14,000 g for 10 min in a cold room (4°C). Then 200 μl supernatant was transferred into a new 1.5 ml microcentrifuge tube, and 800 μl of cool methanol (−80°C) (Fisher Scientific, cat. no. A452SK1) was added to make a final 80% (vol/vol) methanol solution. This mixture was incubated for 8 h at −80°C and then centrifuged at 14,000 g for 10 min (4°C). Subsequently, the supernatant was transferred into a new 1.5 ml microcentrifuge tube, dried in a SpeedVac (Savant AS160, Farmingdale, NY), and stored at −80°C until analysis (21, 22).

Each sample was resuspended in 20 μl of UHPLC-MS grade water (Fisher Scientific, cat. no. MWX00016), and 10 μl per sample was analyzed with UHPLC-MS using the selected reaction monitoring (SRM) method with positive/negative ion polarity switching on the hybrid triple quadrupole/linear ion trap mass spectrometer (AB/SCIEX). A total of 21 amino acids were monitored and detected for each sample. Peak areas from the total ion current for each metabolite SRM transition were integrated using MultiQuant v2.0 software (AB/SCIEX) (21, 22).

### Statistical Analysis

Statistical analysis was carried out in SPSS 14.0. The *T*-test was used to analyze the difference in the continuous variables between the two groups, and the chi-square test was used to evaluate clinical categorical measures (23, 24). The Pearson correlation coefficient was used to calculate the correlations between age and identified metabolites in the two groups. The differences between the two groups were considered significant at *p* < 0.05.

### Metabolomics Data Analysis

As glycine could not be detected in most samples, only 20 amino acids' data were analyzed. All peak areas were aligned and normalized using the median of all amino acids from each sample before further analysis. The normalized data were imported into SIMCA-P version 14.1 (Umetrics Inc., Umea, Sweden) for multivariate analysis, including principal component analysis (PCA) and partial least-squares discriminant analysis (PLS-DA) after mean-centering and unit variance (UV) scaling. The potential biomarkers were filtered and confirmed when their Variable Importance of Projection (VIP) scores are >1 (VIP > 1) (5, 12).

The quality of the PLS-DA model was determined based on a goodness of fit parameter (R2Y) and a goodness of prediction parameter (Q2Y). In addition, the PLS-DA model and the reliability models were further validated using a rigorous permutation test (*n* = 200). The parameters of the models, such as the R2 and Q2 intercepts, were investigated to ensure the quality of the models and to avoid over-fitting (5, 11, 25).

Through analysis of PLS-DA loadings, the metabolites contributing to sample discrimination were identified by Variable Importance of Projection (VIP) scores. The potential biomarkers were filtered and confirmed when their VIP scores are >1 (VIP > 1) (5, 11, 25).

Matlab R2014a (The MathWorks Inc., Natick, MA, USA) was used to perform variable selection of potential biomarkers. The selected potential biomarkers were combined in different combinations for prediction of stroke functional recovery. All prediction combinations were examined separately, using 10-fold cross-validation (26, 27).

In 10-fold cross-validation, nine-tenths of the serum samples from all 40 samples were randomly assigned to the training set. The metabolite profile of this training set was used to diagnose for this prediction task. The remaining one-tenth of the serum samples from all 40 samples formed the test set. This test set was used to validate the metabolite profile diagnostic for the feature of interest. This was repeated 10 times, so that each one-tenth split of the data set acts as the testing set once. Areas under the curve (AUCs) with 95% confidence intervals (CIs) were calculated for sensitivity and specificity values. Mean AUC in the training set, mean AUC in the test set, standard deviation (SD) of AUC in the test set, and 95% CI of mean AUC in the test set were analyzed (26, 27).

Predictive performance results for each prediction combination were compared using area under the receiver operating characteristic curve (AUC of ROC). We note that this predictive performance is for the stratified data set, so our primary interest is in the predictive factors. The values of mean AUC in the test set are regarded as the criteria for selecting the best combination of predictive biomarkers (26, 27). Since the mean test-set AUC scores were used for ranking models and not for formal hypothesis testing, multiple testing corrections were not needed.

## Results

### Clinical Characteristics of Stroke Patients

The clinical characteristics of stroke patients are shown in the Table 1. According to the effectiveness of MRFS, stroke patients were divided into two subgroups: good recovery (GR) group and poor recovery (PR) group. The average age of GR group was significantly lower than that of PR group (61.25 ± 7.84 vs. 71.55 ± 10.39 years) (*p* < 0.001). The time of hospitalization, destination of discharge, total score of FIM at admission and discharge, and effective rate of MRFS and MRFS in the GR group were significantly better than those in the PR group (*p* < 0.001). There was no significant difference in other clinical indexes (including education, BMI, medical history, laboratory items, stroke features, and side of hemiparesis) between the two groups (*P* > 0.05) (Detailed *P*-values can be seen in the Table 1).

**Table 1:** The clinical characteristics of stroke patients.

**Characteristics**	**Good recovery group (*n =* 20)**	**Poor recovery group (*n =* 20)**	***P***
Demographics			
Age(y)	61.25 ± 7.84	71.55 ± 10.39	0.001
Sex, male	8 (40)	10 (50)	0.525
Education			0.525
College	12 (60)	10 (50)	
High school	8 (40)	10 (50)	
Handedness (right)	20 (100)	19 (95)	0.311
BMI	28.48 ± 8.74	29.02 ± 6.69	0.826
Medical history			
Smoking	17 (85)	14 (70)	0.256
Hypertension	13 (65)	17 (85)	0.114
Diabetes	10 (50)	8 (40)	0.525
Hyperlipidemia	6 (30)	9 (45)	0.327
CAD history	1 (5)	5 (25)	0.077
Atrial fibrillation	0 (0)	2 (10)	0.147
Laboratory items			
AST(units/l)	24 ± 13.36	24.9 ± 11.95	0.824
ALT(units/l)	37.25 ± 25.82	39.3 ± 33.81	0.858
BUN(mg/dl)	21.8 ± 18.95	25.55 ± 21.07	0.558
CREAT(mg/dl)	1.05 ± 0.59	0.99 ± 0.45	0.430
HCT (%)	37.25 ± 4.91	35.8 ± 10.23	0.571
Clinical assessment			
Days from onset to admission	8 ± 6.39	8.8 ± 6.55	0.598
Day from onset to blood	12.65 ± 7.02	15.15 ± 8.19	0.307
Length of stay(d)	10.7 ± 3.19	22.9 ± 9.26	0.001
Discharge destination			0.001
Home	20 (100)	4 (20)	
Skilled nursing facility	0 (0)	16 (80)	
Total FIM scores			
Admission	64.20 ± 5.58	42.95 ± 6.65	0.001
Discharge	109.10 ± 6.64	55.45 ± 11.66	0.001
Gain of total FIM (discharge FIM—admission FIM)	40.90 ± 8.29	12.51 ± 9.26	0.001
MRFS	0.71 ± 0.11	0.14 ± 0.10	0.001
MRFS efficiency	0.071 ± 0.023	0.007 ± 0.003	0.001
Stroke features			
Stroke sites			0.598
Supratentorial	18 (90)	19 (95)	
Infratentorial	1 (5)	1 (5)	
Both	1 (5)	0 (0)	
Side of hemiparesis			0.525
Left side	12 (60)	10 (5)	
Right side	8 (40)	9 (45)	
Both side	0 (0)	1 (5)	

*Values shown are as mean ± SD, N (%), or as otherwise indicated*.

*BMI, body mass index; CAD, coronary artery disease*.

### Serum Metabolic Profile of Stroke Patients With Good Recovery and Poor Recovery

**Figure 2:**
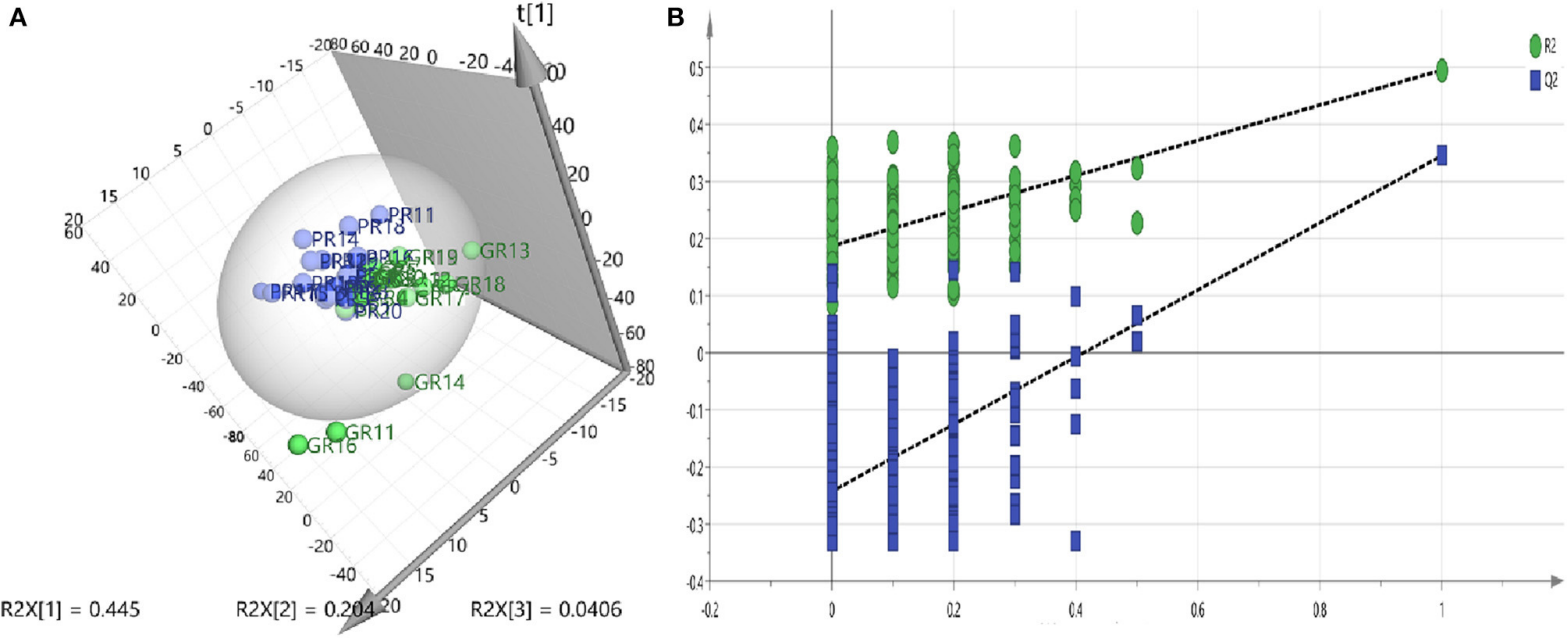
**(A)** PLS-DA models—Two-component plots of GR and PR (green = GR, blue = PR) from UHPLC-MS metabolic profiles, demonstrate clear separation between the GR and PR groups. **(B)** Validation plots for the PLS-DA model obtained from 200 permutation tests (green = R2, blue = Q2): the intercepts of Q2 were <0.

A PLS-DA model was performed to explore the correlation between the GR and PR groups. According to a PLS-DA score plot (Figure 2A), there was a significant separation between stroke patients with good recovery and poor recovery (R2Y = 0.495, Q2Y = 0.345) (Figure 2A), indicating that there is a difference in serum metabolite levels between the GR and PR groups. The PLS-DA model validation was performed using the number of permutations equaling 200 generated and the intercepts of Q2 (fewer than 0), which meant that the PLS-DA model was non-overfitting and reliable (Figure 2B).

### Differences in Metabolites Between Stroke Patients With Good Recovery and Poor Recovery

**Figure 3:**
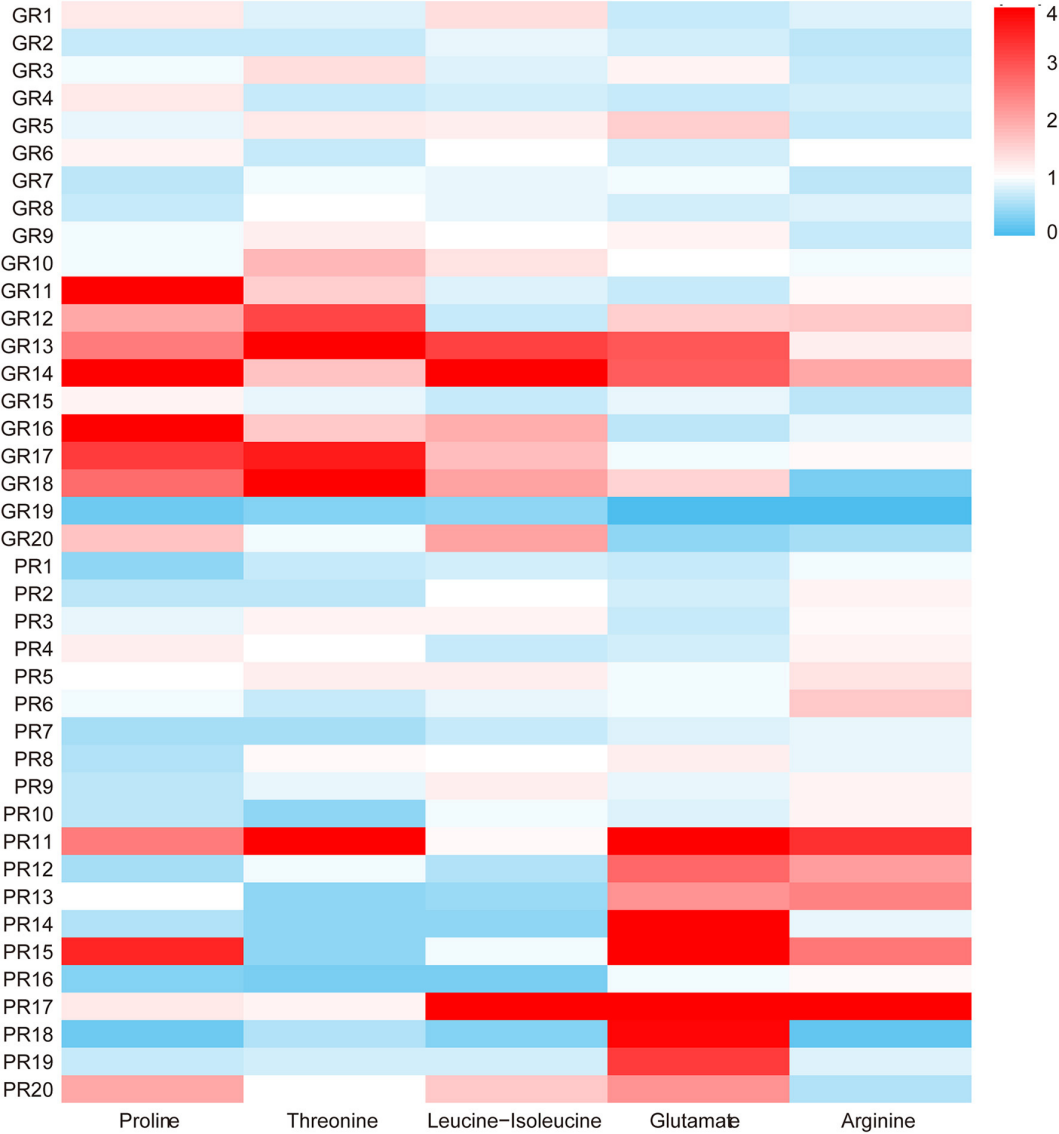
The heat map of the five different metabolites in the GR and PR groups. The colors changing from blue to red indicate the changes of characteristic metabolite content relative to the average normalized quantities of the metabolites in the GR group, blue plots indicate down-regulated metabolites, and red plots indicate up-regulated metabolites.

The PLS-DA model was further analyzed to identify the serum metabolites associated with functional recovery of stroke. Through analysis of PLS-DA loadings, the metabolites contributing to sample discrimination were identified by VIP scores. The potential biomarkers were filtered and confirmed when their VIP scores are >1 (VIP > 1). Through analysis, five serum metabolites [threonine (VIP predicted values: 1.04), arginine (VIP predicted values: 1.47), glutamate (VIP predicted values: 1.89), proline (VIP predicted values: 1.97), and leucine-isoleucine (VIP predicted values: 2.46)] were screened out, which were closely related to the recovery level of stroke (VIP > 1) (Table 2). In contrast to those in the GR group, levels of glutamate (KEGG:C00025) and arginine (KEGG:C00062) were increased in the PR group, whereas levels of leucine-isoleucine (KEGG:C00123), proline (KEGG:C00148), and threonine (KEGG:C00188) were markedly decreased. The average normalized quantities of the differential metabolites in the GR and PR groups are shown in the heat map (Figure 3).

**Table 2:** Results of the VIP predicted values of differential metabolites between stroke patients with GR and PR.

**Metabolites**	**VIP predicted values**	**Fold change**
Leucine-isoleucine	2.46	0.65
Proline	1.97	0.54
Glutamate	1.89	2.11
Arginine	1.47	1.74
Threonine	1.04	0.66

*FC, Fold change, ratio of metabolite's relative peak area in PR stroke patients to that of GR stroke patients*.

*This table shows those metabolites with a VIP predicted value >1 and their fold change. When the fold change is >1, the metabolite levels in the PR group were higher than those in the GR group. When the fold change is <1, metabolite levels in the PR group were lower compared with those in the GR group*.

### Identify Potential Predictive Biomarkers in Serum

Models were fit where five metabolites were considered in all possible combinations, including one single metabolite combination, two different metabolites in combination, and up to the combination of all five metabolites (Table 3). There were 31 combinations, and all combinations of the selected five metabolites for prediction of functional recovery after stroke were analyzed by cross-validation (Table 3).

**Table 3:** Results of 10 independent replicates of 10-fold cross-validation of all combinations of the selected five metabolites for prediction of functional recovery after stroke.

**NO**	**Proline**	**Threonine**	**Leucine-Isoleucine**	**Glutamate**	**Arginine**	**Mean AUC in training set**	**Mean AUC in test set**	**SD of AUC in test set**	**95% CI of mean AUC in test set**
1	1	0	0	1	1	0.952	0.835	0.109	(0.756, 0.913)
2	1	0	1	1	1	0.956	0.817	0.113	(0.735, 0.897)
3	1	0	0	0	1	0.920	0.808	0.099	(0.737, 0.879)
4	0	1	0	1	1	0.947	0.799	0.115	(0.717, 0.881)
5	0	1	1	1	1	0.991	0.795	0.094	(0.727, 0.861)
6	0	0	1	1	1	0.924	0.793	0.106	(0.716, 0.868)
7	1	1	0	1	1	0.972	0.792	0.130	(0.699, 0.884)
8	1	0	1	0	1	0.929	0.788	0.093	(0.721, 0.854)
9	1	1	0	0	1	0.929	0.773	0.114	(0.691, 0.853)
10	1	1	1	0	1	0.935	0.771	0.114	(0.689, 0.852)
11	1	0	0	1	0	0.870	0.767	0.115	(0.684, 0.849)
12	0	0	1	0	1	0.857	0.766	0.094	(0.698, 0.833)
13	1	0	1	1	0	0.871	0.758	0.103	(0.684, 0.831)
14	1	1	1	1	1	0.994	0.758	0.119	(0.672, 0.843)
15	0	1	1	0	1	0.895	0.744	0.120	(0.658, 0.830)
16	1	1	0	1	0	0.909	0.744	0.136	(0.647, 0.842)
17	1	1	1	1	0	0.908	0.728	0.121	(0.642, 0.815)
18	0	0	0	1	1	0.846	0.723	0.096	(0.655, 0.792)
19	0	1	0	0	1	0.852	0.713	0.102	(0.640, 0.787)
20	0	1	1	1	0	0.877	0.698	0.134	(0.602, 0.794)
21	0	0	0	0	1	0.758	0.698	0.084	(0.637, 0.758)
22	0	1	0	1	0	0.850	0.694	0.128	(0.603, 0.785)
23	1	0	0	0	0	0.745	0.654	0.115	(0.572,0.737)
24	0	1	0	0	0	0.750	0.653	0.130	(0.561, 0.746)
25	1	1	0	0	0	0.748	0.628	0.129	(0.536,0.721)
26	0	0	1	1	0	0.764	0.628	0.092	(0.561,0.694)
27	0	0	0	1	0	0.680	0.626	0.097	(0.557,0.695)
28	1	0	1	0	0	0.743	0.625	0.111	(0.546, 0.704)
29	1	1	1	0	0	0.751	0.604	0.119	(0.519, 0.689)
30	0	1	1	0	0	0.730	0.573	0.126	(0.483, 0.664)
31	0	0	1	0	0	0.645	0.536	0.102	(0.463, 0.609)

*1 refers to containing this metabolite when performing 10-fold cross-validation, and 0 means that the metabolite is not contained*.

*According to the metabolites combinations, there were 31 combinations for prediction of functional recovery after stroke that were analyzed by cross-validation. The standard deviations (SD) of the AUC in the testing set are between 0.09 and 0.13, which indicates the model of this study is steady. The values of mean AUC in the test set are used as criteria for evaluation. The panel including proline, glutamate and arginine had the highest values of mean AUC in the test set [0.835, 95% CI (confidence interval): (0.756, 0.913)] [mean AUC in the training set:0.952, 95% CI: (0.951, 0.953)] in the 31 combinations. As a result, this panel was the best combination of predictive biomarkers of the 31 combinations*.

**Figure 4:**
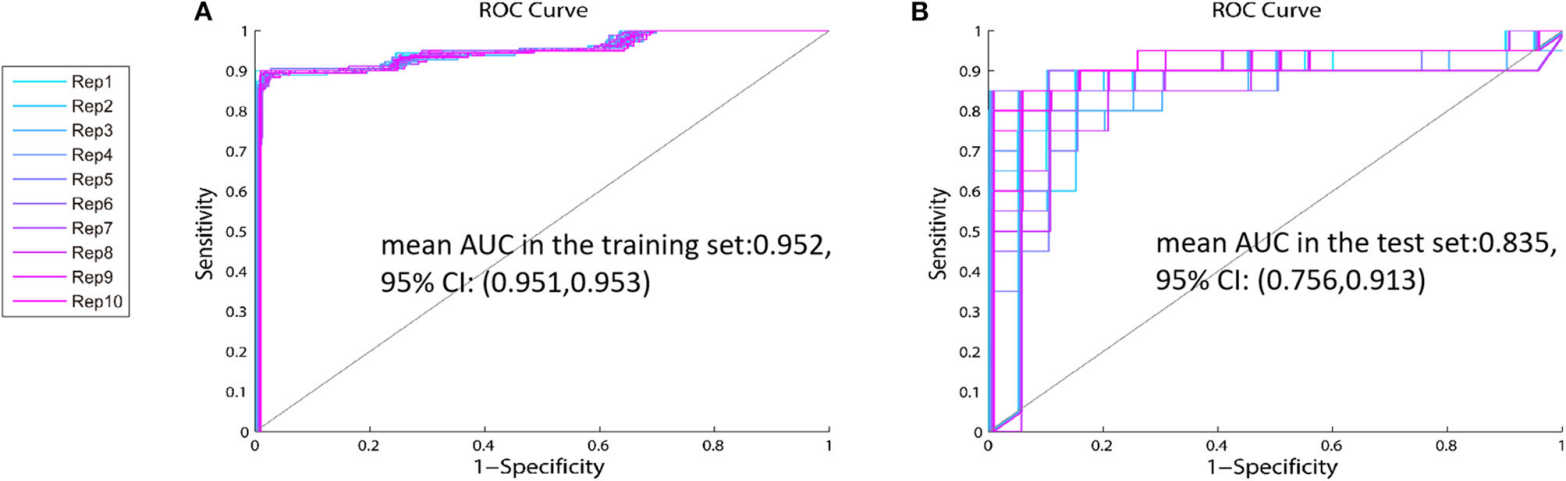
The ROC curve of the identified panel—The values of mean AUC of the panel including proline, glutamate, and arginine are 0.952 (95% CI: 0.756, 0.913) in the training set **(A)**, and 0.835 (95% CI: 0.951, 0.953) in the test set **(B)**.

Cross-validation is a well-known technique to choose tuning parameters of a model, while limiting the risk of overfitting (26, 27). As shown in Table 3, standard deviations (SD) of the AUC in the testing set are between 0.09 and 0.13, which indicate that the model is steady. Single metabolite did not offer good predictive value but displayed low sensitivity and specificity in both the training and test sets. The panel including proline, glutamate, and arginine had the highest values of mean AUC in the test set [0.835, 95% CI: (0.756, 0.913)] and mean AUC in the training set: [0.952, 95% CI: (0.951, 0.953)] of the 31 combinations (Table 3, Figure 4). Therefore, this panel was regarded as the best combination of predictive biomarkers out of the 31 combinations.

As shown in Table 3, arginine was in all of the top 10 combinations ranked by values of mean AUC in the test set. Four out of the top five combinations ranked by values of mean AUC in the test set contained both glutamate and arginine. When leucine-isoleucine, threonine, and proline were combined with arginine, these new panels all had high predictive value for stroke functional recovery (Table 3). The models suggest that arginine is important in stroke recovery.

### The Correlations Between Age and Metabolites

**Figure 5:**
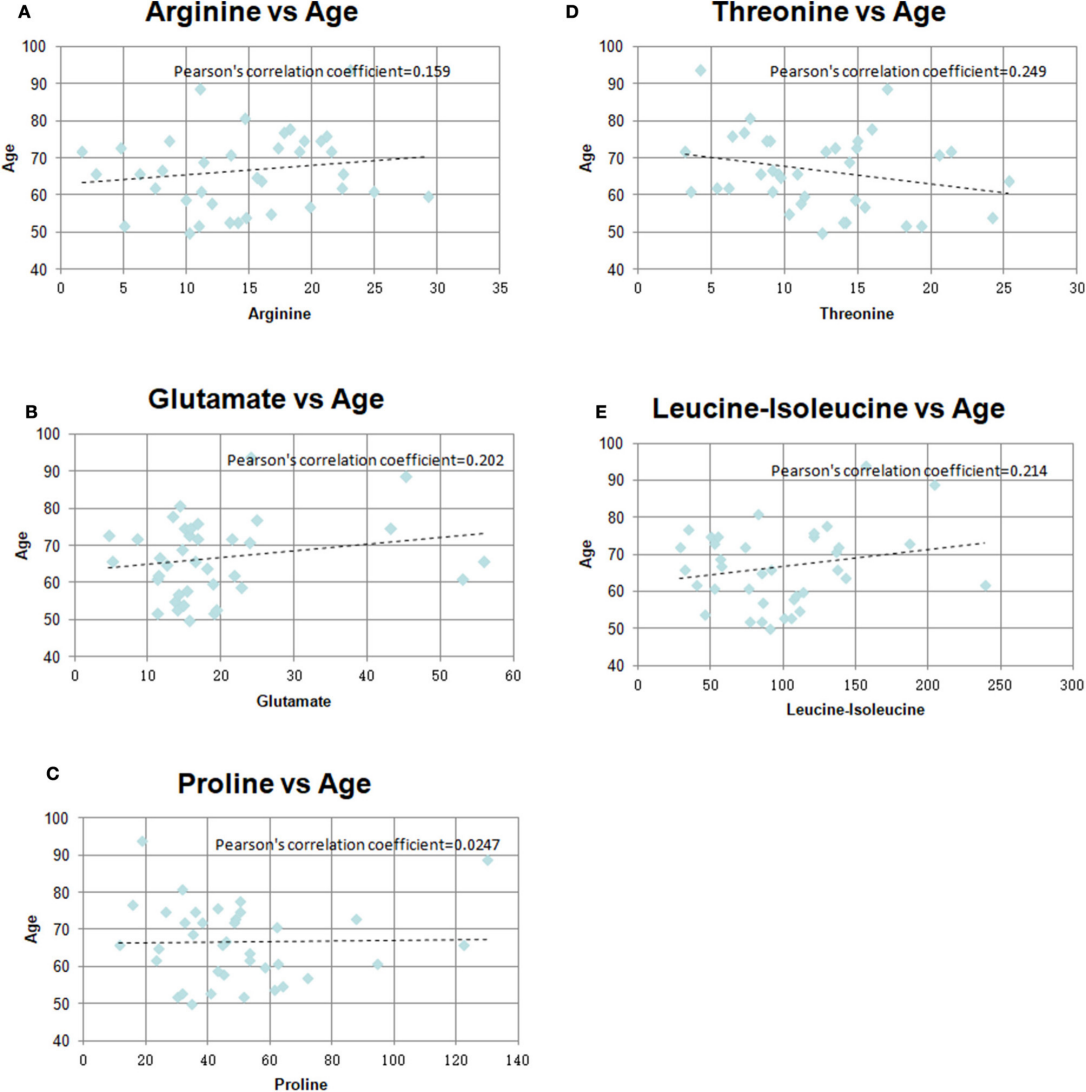
Pearson's correlation coefficients graph between age and identified metabolites in the two groups **(A)** Arginine vs. Age, **(B)** Glutamate vs. Age, **(C)** Proline vs. Age, **(D)** Threonine vs. Age, **(E)** Leucine-isoleucine vs. Age.

There were significant differences in age between the GR and PR groups. So, we investigated the correlation between age and identified metabolites in the two groups. As shown in Table 4 and Figure 5, there was little significant correlation between identified metabolites and age in the two groups.

**Table 4:** The Pearson's correlation coefficients between age and identified metabolite.

	**Pearson's correlation coefficients**
Correlation between of age and arginine	0.159
Correlation between of age and glutamate	0.202
Correlation between of age and proline	0.024
Correlation between of age and threonine	0.249
Correlation between of age and leucine-isoleucine	0.214

**Figure 6:**
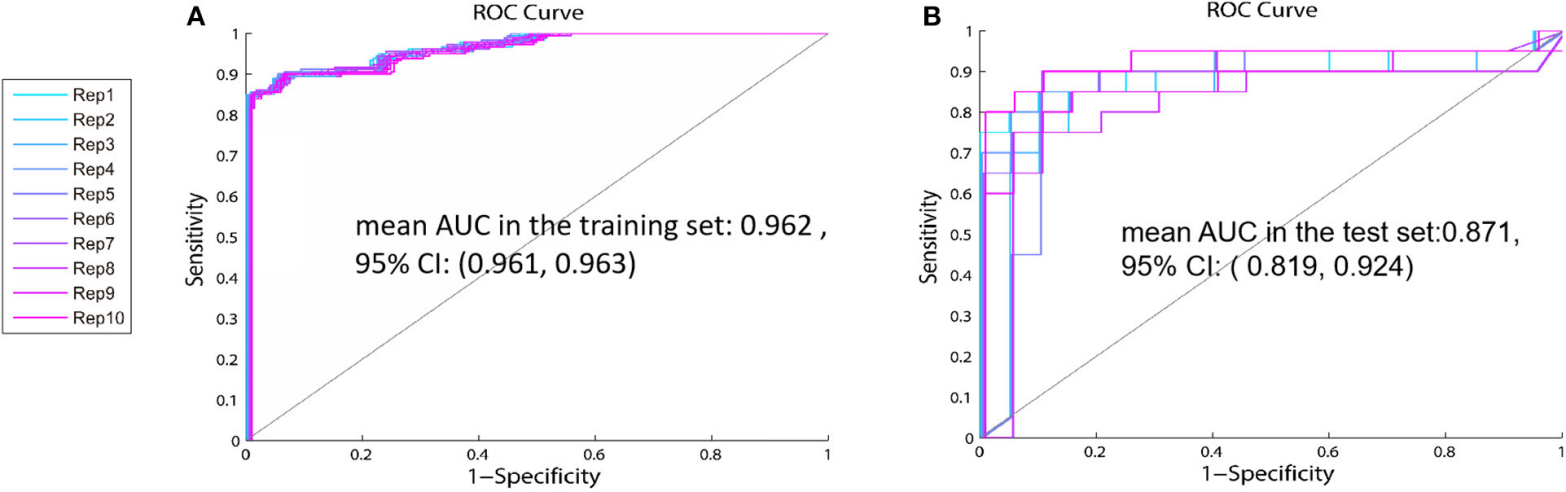
The ROC curve of the 4-variable panel including age, proline, glutamate, and arginine—The values of mean AUC of the panel including age, proline, glutamate, and arginine in the training set **(A)** is 0.962 (95% CI:0.961, 0.963) and 0.871 (95% CI:0.819, 0.924) in the test set **(B)**.

Based on the above results, we recombined the age and the panel of proline, glutamate, and arginine. We analyzed the 4-variable panel, respectively, in the training and test sets. The values of mean AUC of the 4-variable panel was 0.962 (95% CI: 0.961, 0.963) in the training set, and 0.871 (95% CI: 0.819, 0.924) in the test set, which had higher sensitivity and specificity distinguished between patients with GR and PR compared with the identified panel from 31 combinations (Figure 6).

## Discussion

Amino acids are among the most important disturbed metabolites after stroke, but the association of amino acid levels with level of stroke recovery is not clear. Our main findings were that there was a significant difference in leucine-isoleucine, proline, threonine, glutamic acid, and arginine levels between the GR and PR groups. The panel of combined proline, glutamate, and arginine provided high sensitivity and specificity in prediction of functional recovery.

**Figure 7:**
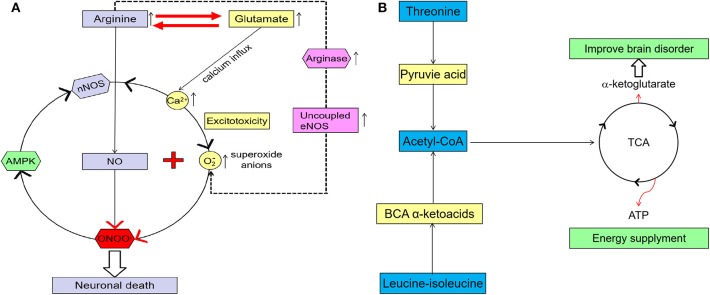
**(A)** A higher glutamate concentration in the brain can induce excitotoxicity, and the increase of Ca2+ concentrations in the cell, which may cause mitochondrial dysfunction and increase oxidative stress and neuron death. A high arginine concentration can increase arginase activity, which leads to an uncoupling of eNOS and induces more neurotoxic substance (imaginary line). Excessive glutamate has excitotoxicity, and arginine can also increase oxidative stress and induce more neurotoxins by itself. Arginine and glutamate can be converted into each other and linked in their metabolism. When higher concentrations of arginine and glutamate come together, more neurotoxins such as peroxynitrite are produced. Adenosine monophosphate activated protein kinase (AMPK) also can be activated by peroxynitrite, so nNOS, peroxynitrite, and AMPK become one vicious cycle. All these will ultimately lead to neuronal death and functional impairment. **(B)** Both leucine-isoleucine and threonine can be converted to acetyl-coA and come into the TCA). TCA can provide ATP and AKG for the brain, which have an important effect on brain function recovery after stroke.

Our results showed that arginine levels in the PR group were significantly higher than those in the GR group. This is in line with evidence suggesting that high arginine concentration can induce neurotoxic substances (12, 14, 28). *S*tudies have shown that arginine in plasma can cross the blood-brain barrier (BBB) into the brain (16, 28), and that there is a high correlation between serum arginine and arginine in cerebrospinal fluid (CSF) in stroke patients (16). As shown in the arginine metabolic pathway (Figure 7A), arginine is the precursor of nitric oxide (NO), and NO is synthesized from L-arginine by nitric oxide synthase (NOS), which includes neuronal NOS (nNOS), endothelial NOS (eNOS), and inducible NOS (iNOS). In the human brain, the synthesis of NO is mainly related to nNOS that lies in neurons (13). At physiological concentrations (EC_50_ 1–4 nM) (29, 30), NO can regulate blood flow, relax blood vessels, and inhibit platelet aggregation, which are beneficial for the recovery of stroke dysfunction (28, 31). Several studies have shown that arginine supplementation contributes to stroke recovery, which is related to the PKC-mediated NO signaling pathway (32). However, recent studies indicate that high concentrations of arginine can increase oxidative stress in the general population (28). Arginase (also present in the brain) is an enzyme that catalyzes the conversion of arginine to ornithine, competing with eNOS (in the choroid plexus and vascular endothelium) for arginine (28). A high concentration of arginine could stimulate the expression and activation of arginase, and the maximal catalytic activity of arginase is higher than that of eNOS (15, 28). The increase of arginase activity will decrease the catalytic activity of arginine for NO production and lead to the uncoupling of eNOS, while uncoupled eNOS can induce the transfer of electrons from NADPH to oxygen molecules to form superoxide anions (${\text{O}}_{2}^{-}$). *In vitro* studies also found that a high concentration of arginine decreased the antioxidant capacity of brain tissue, which in turn increased oxidative stress, inhibited the activity of glutathione peroxidase in the brain tissue, induced the production of neurotoxic substances, and finally decreased the recovery of nerve function (15, 28).

In this study, in addition to arginine, serum glutamate levels in patients with PR were also significantly higher than those in patients with GR. Glutamate is one of the most abundant free amino acids in the mammalian central nervous system (CNS) and is at the intersection of multiple metabolic pathways (33). In its physiological concentration, glutamate is crucial for various physiological processes, particularly synaptic transmission. But a higher glutamate concentration in the brain may trigger secondary brain injury following acute ischemic stroke, including neuron death, axonal injury, and mitochondrial dysfunction (34, 35). First of all, neuronal death, impaired energy supply, and increased oxidative stress caused by mitochondrial deregulation are not conducive to functional recovery after stroke. Secondly, glutamate receptors expressed on brain endothelial cells play an important role in regulating the function of the BBB (36). The excessive activation of glutamate receptors may lead to the abnormal expression and distribution of tight junction proteins in endothelial cells, resulting in the destruction of the BBB (36). In this case, the harmful substances may easily pass through the BBB and aggravate the death and injury of neurons. Thirdly, abnormal synaptic transmission induced by a high concentration of glutamate may decrease the synaptic plasticity and inhibit the recovery of neural function after stroke (Figure 6A) (36).

High concentrations of arginine and glutamate may lead to stronger neuroexcitotoxicity through synergistic action (37, 38). nNOS is Ca^2+^-dependent and will be activated to generate NO when calmodulin forms Ca^2+^/calmodulin complex with calcium (37). During the ischemia reperfusion (IR) phase of ischemic stroke, N-methyl-D-aspartic acid (NMDA) receptor-mediated excitotoxicity caused by increased glutamate levels may lead to calcium dysregulation and stimulate nNOS to produce more NO in the neurons (35). At the same time, this excitotoxicity may also trigger mitochondrial dysfunction and reduce the ability of mitochondria to resist oxidative stress, resulting in more reactive oxygen species (38, 39). Under oxidative stress, the production of S-nitrosoglutathione (GSNO) will be decreased, and the superoxide anion will react with NO to form a peroxynitrite anion (ONOO^−^), which is of high neurotoxicity (15, 40). ONOO^−^ can inhibit the activity of cytochrome c oxidase in the respiratory chain and destroy the electron transport-associated proteins in mitochondria, which leads to energetic failure and neuron death (15, 40). With the death of neurons and mitochondria, the oxidative stress levels in the brain may increase and lead to the production of more neurotoxic substances, such as nitrotyrosine (Figure 7A).

It was found that NMDA-receptor-mediated calcium dysregulation can activate nNOS by re-modifying its chemical structure, such as phosphorylation of Ser1412 (40). Adenosine monophosphate activated protein kinase (AMPK) is one energy sensor with a high expression of neurons (41). AMPK is activated when cellular energy is decreasing. The activation of AMPK may keep nNOS in a hyperactivated state via sustained phosphorylation of Ser1412 (40, 41). However, AMPK can also be activated by peroxynitrite, resulting in a vicious circle of nNOS, peroxynitrite, and AMPK (Figure 6A) (40). All of these will ultimately lead to neuronal death and functional impairment. Therefore, glutamate and arginine, both of which have high sensitivity and specificity to functional results, appeared four times in the top five combinations sorted according to the average value of AUC. This result is consistent with the synergistic effect of excessive glutamate and arginine on neurotoxicity. As shown in Figure 6A, arginine is also the precursor to glutamate. Therefore, an increase in arginine can cause an increase in glutamate, and arginine itself may also trigger this synergistic effect. This may partly explain why arginine could be found in the top ten combinations in the test set.

The levels of serum leucine-isoleucine and threonine in the PR group were significantly lower than those in the GR group. Studies have shown that leucine-isoleucine plays an active role in inhibiting an excessive glutamate concentration and excitotoxicity induced by stroke (5), and it is also an important component of energy metabolism (42). Threonine is also a fuel substrate that can be converted to pyruvic acid and become part of the tricarboxylic acid cycle (TCA) (43). TCA can provide adenosine triphosphate (ATP) and æ-ketoglutarate to the brain, while reducing leucine-isoleucine and threonine leads to a reduction in ATP and æ-ketoglutarate. The brain is an organ that consumes a lot of energy without storing any, so an abnormal energy metabolism may cause a brain disorder. æ–ketoglutarate is an important intermediate metabolite in the TCA cycle that can also improve ischemia-induced brain disorders (Figure 7B) (44).

The relationship between proline and stroke is unclear. It has been reported that the level of serum proline in patients with acute ischemic stroke is significantly lower than that in normal controls (7). He et al. further proved that the level of serum proline in the PR group was significantly lower than that in the GR group, which may be related to the fact that proline can enhance the stability of proteins and cell membranes (45).

Our results revealed that the serum levels of five amino acids (leucine-isoleucine, proline, threonine, glutamic acid, and arginine) differed significantly between GR and PR groups. The panel grouped by proline, glutamate, and arginine had the highest sensitivity and specificity in predicting recovery and functional outcomes of stroke. This finding suggests that the metabolomic process combined high level of neuroexcitoxicity with low level of cell membrane stability may worsen stroke recovery.

## Study Limitations

The stratification method used in this study enhanced the power of detection by targeting the extremes; however, this study is still limited by relatively small sample size. Second, subjects from this study were from one single, urban rehabilitation hospital; therefore, the findings cannot be generalized to the general population. Third, in this study, the average age of the GR group was significantly younger than the average age of the PR group (*p* < 0.05). The age difference may contribute to the difference in metabolimic profiling. In addition, the age difference could also cause bias of the results. Further study with age-matched groups, multi-center and large sample size is warranted to confirm the findings.

## Conclusion

1. High levels of glutamate and arginine are associated with PR after stroke, which is related to oxidative stress and excitotoxicity.
2. Leucine-isoleucine, threonine, and proline involved in energy metabolism are positively related to functional recovery after stroke.
3. Reduced leucine-isoleucine, threonine, and proline, as well as all or only one or two of them, were combined with elevated arginine into new panels, and these new panels illustrate a high predictive value for stroke functional recovery. The panel grouped by proline, glutamate, and arginine has the highest sensitivity and specificity on predicting recovery and functional outcomes of stroke of all 31 combinations. Age can increase sensitivity and specificity of this identified panel for stroke functional recovery.

## Data Availability Statement

The datasets generated during and/or analyzed during the current study are available from the corresponding author on reasonable request.

## Ethics Statement

The study was approved by the ethics committee of Spaulding Rehabilitation Hospital. Written informed consent was not required as per local legislation and national guidelines.

## Author Contributions

XW, TL, and QW contributed to the inception and design of the paper and writing of the manuscript. HS, SC, GL, AC, and PF contributed to patient recruitment and data collection. XW, QW, XL, and LW contributed to the statistical analysis and research design. All authors approved the final version of the manuscript.

### Conflict of Interest

The authors declare that the research was conducted in the absence of any commercial or financial relationships that could be construed as a potential conflict of interest.

## Abbreviations

AMPK: adenosine monophosphate activated protein kinase
AUC: area under the receiver operating characteristic curve
CSF: cerebrospinal fluid
FIM: Functional Independence Measure
GR: good recovery
IR: ischemia reperfusion
MRFS: Montebello Rehabilitation Factor Score
NO: nitric oxide
NOS: nitric oxide synthase
PCA: principal component analysis
PLS-DA: partial least-squares discriminant analysis
PR: poor recovery
ROC: receiver operating characteristic
TCA: tricarboxylic acid cycle
UHPLC-MS: ultra high performance liquid chromatography—mass spectrometry
UV: unit variance
VIP: variable importance of projection.
